# A comparative analysis of YOLOv8 and nnU-Net v2 based pipelines for sex and age estimation from maxillary sinus morphometry on panoramic radiographs

**DOI:** 10.1007/s00414-026-03825-x

**Published:** 2026-05-09

**Authors:** Mert Ocak, Cumali Çatak, Seçil Aksoy, Kaan Orhan

**Affiliations:** 1 Department of Basic Medicine Science- Anatomy, Faculty of Dentistry, Ankara University, Türkiye Ankara, Turkey; 2 Department of Forensic Anthropology, Graduate School of Health Sciences, Ankara University, Ankara, Türkiye Turkey; 3 Department of Forensic Anthropology, Institute of Forensic Sciences, Ankara University, Ankara, Türkiye Turkey; 4 Department of Oral and Maxillofacial Radiology, Faculty of Dentistry, Near East University, Mersin, Türkiye; 5 Department of Oral and Maxillofacial Radiology, Faculty of Dentistry, Ankara University, Ankara, Türkiye

**Keywords:** Maxillary sinus, Deep learning, YOLOv8, nnU-Net, Sex estimation, Age estimation

## Abstract

**Objective:**

This study aimed to develop and compare two deep learning-based segmentation–radiomics pipelines — YOLOv8-Hybrid and nnU-Net v2 — for automated sex classification and age estimation from maxillary sinus morphometry on panoramic radiographs.

**Methods:**

A balanced dataset of 1,024 panoramic radiographs (512 males, 512 females; age 18–81 years) was collected from Near East University, North Cyprus. Ground truth sinus annotations were generated by an expert oral radiologist and validated through dual-annotator inter-observer reliability assessment (ICC (2,1) = 0.94–0.97). The YOLOv8-Hybrid pipeline employed YOLOv8n-seg coarse segmentation, U-Net boundary refinement, > 120 morphometric and radiomic features, and CatBoost/XGBoost classifiers. The nnU-Net v2 pipeline used auto-configured 2D U-Net segmentation with identical feature extraction and XGBoost prediction. Both pipelines underwent 5-fold cross-validation with patient-level splitting, transfer learning, Bayesian hyperparameter optimization, and SHAP interpretability analysis.

**Results:**

nnU-Net v2 achieved statistically significant superiority in sex classification (AUC = 0.927 [95% CI: 0.881–0.964]) over YOLOv8-CatBoost (AUC = 0.893 [0.841–0.938]; DeLong *p* = 0.024, Cohen’s d = 0.48). Both pipelines demonstrated comparable age estimation performance (MAE ≈ 7.2 years). YOLOv8 showed exceptional consistency (mAP@50 = 98.19%, CV = 0.77%). SHAP analysis identified bilateral area difference as the most determinant feature (sex: 0.42, age: 0.51). External validation on 50 independent images confirmed model generalizability.

**Conclusions:**

This study provides the first systematic comparison of YOLOv8 and nnU-Net v2 for forensic maxillary sinus analysis. nnU-Net v2 is recommended for precision-critical forensic reporting, while YOLOv8-Hybrid is suited for high-throughput screening. The > 120 radiomic/morphometric features establish a comprehensive framework for automated biological profiling.

## Introduction

Forensic anthropology aims to determine the biological profile (sex, age, stature, ancestry) of unidentified human remains [1]. In the forensic context, the term “sex” refers to biological sex inferred from skeletal morphology and should not be confused with gender identity. Sex and age estimation play critical roles in mass disasters, war crimes, and forensic investigations by narrowing the search universe [2, 3]. Classical anthropometric approaches provide sex determination with high accuracy from the pelvis (90% accuracy) and cranial morphology (80–85% accuracy) [4]. Dental development [5], epiphyseal fusion, and pubic symphysis changes are considered the gold standard for age estimation [6]. However, in mass disasters, trauma, and advanced decomposition, these structures may be damaged [7, 8]. Analysis of protected anatomical regions through objective, reproducible, and automated methods is therefore necessary.

Maxillary sinuses carry forensic biomarker potential as durable structures resistant to postmortem changes in the skull [9, 46, 47]. Larger sinus volumes after puberty due to androgenic effects in males have been reported [10–12]. Biological sexual dimorphism in size and shape has been demonstrated in numerous studies. Panoramic radiography is used for both clinical and forensic purposes with low radiation dose (0.002–0.010 mSv; ~1% of CT) and widespread accessibility [13]. Radiomic textural metrics (gray-level co-occurrence matrix, gray-level run-length matrix) enhance age and sex signals by quantitatively capturing tissue heterogeneity [14, 15].

Deep learning has created a paradigm-shifting impact in medical image analysis [16]. Convolutional neural networks, including deep residual architectures [27], automatically learn complex patterns [17, 57]. Their application in forensic science enables automated biological profiling from skeletal remains [18]. Two different paradigms are prominent in deep learning methodology: the YOLO family provides high speed with single-stage detection and segmentation [19–21]; nnU-Net is accepted as the gold standard in medical segmentation [22, 23].

Previous studies on age estimation and sex classification from the maxillary sinus have mainly relied on manual linear/volumetric measurements and discriminant function analysis [10, 11, 28, 55, 60]. In contrast, recent years have reported high accuracy from machine learning/deep learning-based approaches for automated age and sex estimation from panoramic radiographs [29, 31, 43].

The unique aspect of this study is to develop and compare two deep learning-based segmentation–radiomics pipelines within the same methodological framework for age estimation and sex classification using maxillary sinus morphology from panoramic radiographs:

1) YOLOv8-hybrid: YOLOv8n-seg segmentation + U-Net boundary refinement + > 120 features + CatBoost.

2) nnU-Net v2: auto-configured 2D U-Net + identical features + XGBoost.

Hypotheses: (H1) nnU-Net v2 is superior in pixel segmentation (median DSC > 0.85); (H2) YOLOv8 is superior in speed and consistency (mAP > 98%, CV < 1%); (H3) features extracted from nnU-Net masks provide higher accuracy in sex/age prediction (AUC > 0.92); (H4) bilateral area difference provides the highest contribution in SHAP analysis (> 0.40). These thresholds were established a priori based on published benchmarks: DSC > 0.85 corresponds to “good” segmentation performance as reported in the Medical Segmentation Decathlon [22] and recommended by recent validation guidelines [54]; mAP > 98% aligns with YOLO-based dental segmentation benchmarks [21, 51]; AUC > 0.92 reflects the upper range of previously reported forensic sinus classification studies [11, 48, 60]; and SHAP > 0.40 was set to identify features with clinically meaningful contributions exceeding random baseline levels.

## Materials and methods

### Study design and ethical approval

This study, designed as a retrospective cohort study, was approved by the Near East University Scientific Research Ethics Committee and was conducted in accordance with the principles of the Declaration of Helsinki (decision no. YDU/2025/139–2045, dated 05.01.2026). Due to the retrospective design of the study and the complete de-identification of the data, the requirement for patient consent was waived in accordance with ethics committee standards. Panoramic radiographs taken for routine dental treatment from the archives of Near East University Faculty of Dentistry, North Cyprus, between 2011 and 2013, were used for secondary forensic research purposes. To protect patient privacy, all images were completely anonymized, and demographic information (age, sex) was stored in a separate secure database (labels.csv). All analyses were conducted on de-identified data. This study was reported in accordance with the STROBE (Strengthening the Reporting of Observational Studies in Epidemiology) and TRIPOD-AI (Transparent Reporting of a multivariable prediction model for Individual Prognosis Or Diagnosis — Artificial Intelligence) guidelines (completed checklists available as supplementary material upon request).

### Study population

Inclusion criteria were defined as: being 18 years of age or older, having images of diagnostic quality without significant artifacts, and having no history of pathology, trauma, or surgery affecting the maxillary sinus region. Individuals with artifacts degrading image quality, pathological lesions preventing assessment of sinus boundaries, or a history of maxillofacial surgery were excluded from the study.

In accordance with these criteria, a balanced dataset consisting of panoramic radiographs from a total of 1,024 patients was created. The sample includes 512 males (50%) and 512 females (50%). A priori power analysis (G*Power 3.1, two-tailed, α = 0.05, power = 0.80, medium effect size d = 0.50) indicated a minimum of 128 subjects per group; the present sample (512 per group) provides > 99% statistical power. The age range is 18–81, with a mean age of 49.5 ± 18.5 years (Table 1). The study population represents a North Cyprus-based university hospital population; it consists predominantly of Mediterranean-origin (Turkish-Cypriot, 78%) adult patients. Chronological age estimation (hereafter referred to as “age estimation”) was used in the study; the standard practice in forensic anthropology for biological profiling is the training and validation of models with chronological age based on registry documents [3, 6]. To eliminate inter-device variability, all images were obtained with the same Planmeca Proline CC (Helsinki, Finland) panoramic X-ray device using a standard protocol (70–77 kVp, 8–10 mA, 12–18 s, focal point-detector distance: 1.5 m).

**Table 1:** Demographic distribution of the study population and dataset split characteristics

Characteristic	Total (*n* = 1024)	Training (*n* = 717)	Validation (*n* = 154)	Test (*n* = 153)
Number of patients	1024	717 (70.0%)	154 (15.0%)	153 (15.0%)
Sex - Male	512 (50.0%)	358 (49.9%)	77 (50.0%)	77 (50.3%)
Sex - Female	512 (50.0%)	359 (50.1%)	77 (50.0%)	76 (49.7%)
Age Mean ± SD	49.5 ± 18.5	49.3 ± 18.4	49.8 ± 18.7	50.1 ± 18.6
Age Median [IQR]	48 [33–65]	48 [33–65]	49 [34–66]	49 [34–65]
Age Min - Max	18–81	18–81	19–81	18–80
Age 18–33 years	256 (25.0%)	179 (25.0%)	39 (25.3%)	38 (24.8%)
Age 34–49 years	256 (25.0%)	179 (25.0%)	38 (24.7%)	39 (25.5%)
Age 50–65 years	256 (25.0%)	180 (25.1%)	38 (24.7%)	38 (24.8%)
Age 66–81 years	256 (25.0%)	179 (25.0%)	39 (25.3%)	38 (24.8%)

### Image preprocessing and ground truth generation

To optimize the performance of deep learning models, a standard preprocessing pipeline was applied to all images.

#### Physical Calibration and Spatial Resolution

Images in original BMP format are 16-bit depth and average 2000 × 1000 pixels in size. The Planmeca Proline CC device has a pixel size of 0.079 mm/pixel, with a corresponding pixel area of (0.079 mm)² ≈ 0.006241 mm²/pixel. This calibration was used in converting all morphometric measurements (area, perimeter, height, width) from pixel values to millimetric physical dimensions [45]. Images were not resized; original spatial resolution was preserved.

#### Intensity Normalization

Contrast Limited Adaptive Histogram Equalization (CLAHE) algorithm was applied to make the faint boundaries of sinuses more distinct (clipLimit = 2.0, tileGridSize=(8, 8)). CLAHE provides local contrast enhancement while maintaining global histogram balance. Subsequently, images were converted to 8-bit PNG format (min-max normalization in the 0–255 range) to provide a standard input format for model training. For all models, intensity values were scaled to the [0,1] range using min-max normalization rather than z-score standardization.

Radiomic Feature Quantization Parameters: For radiomic feature extraction with PyRadiomics (version 3.0.1) [61, 62], the following standardization parameters were used: gray-level binWidth = 25 (10 bins in 8-bit intensity range), no intensity rescaling (already in [0,255] range), no voxel spacing resampling (2D image, non-isotropic panoramic geometry). All radiomic calculations were performed only within the segmented sinus ROI.

### Ground truth annotation and inter-observer reliability

After preprocessing, the right and left maxillary sinuses in the entire dataset (1,024 images) were meticulously hand-labeled as polygons using the LabelMe v5.0.1 tool by a board-certified oral and maxillofacial radiologist with more than 15 years of experience (Observer 1: S.A.). These expert labels were accepted as “ground truth” for training and evaluation of models.

To assess inter-observer reliability, a randomly selected subset of 100 images (stratified by sex and age group, random seed = 42) was independently re-annotated by a second experienced observer (Observer 2: K.O., Professor of Oral Radiology with > 20 years of experience). The two observers were blinded to each other’s annotations. Inter-observer agreement was evaluated using the following metrics:

- (a) Intraclass Correlation Coefficient (ICC): Two-way random-effects model, single measures, absolute agreement (ICC (2,1)) was calculated for morphometric measurements (area, perimeter, height, width) extracted from annotations of both observers
- (b) Dice Similarity Coefficient (DSC): Pixel-level overlap between Observer 1 and Observer 2 annotations was computed for each sinus
- (c) Bland-Altman Analysis: Systematic bias and 95% limits of agreement between observers were assessed for sinus area measurements.

Results demonstrated excellent inter-observer reliability (Table 8):

- Sinus area: ICC = 0.968 [95% CI: 0.954–0.978], mean DSC = 0.934 ± 0.028.
- Sinus perimeter: ICC = 0.951 [95% CI: 0.932–0.966].
- Sinus height: ICC = 0.943 [95% CI: 0.921–0.960].
- Sinus width: ICC = 0.957 [95% CI: 0.940–0.970].

All ICC values exceeded 0.90, indicating “excellent” agreement according to the Cicchetti (1994) classification [50]. Bland-Altman analysis revealed no systematic bias (mean difference: −0.8 mm² [95% LoA: −22.1, 20.5 mm²] for sinus area). These results confirm the reliability of the ground truth annotations used in this study.

### Experimental setup and methodological framework

Data Leakage Prevention: Preventing data leakage is critical to accurately evaluating the model’s generalization performance. For this purpose, the dataset was split according to patient identification numbers. This patient-level split guarantees that all data from one patient is in only one set (training, validation, or test). The dataset was divided into 70% training (717 patients), 15% validation (154 patients), and 15% test (153 patients) using the StratifiedGroupKFold method, which ensures that age and sex are preserved in each set. Split validation: It was verified that there was no patient-level overlap (no data leakage) between training, validation, and test sets.

#### Data Augmentation

To prevent overfitting and increase the robustness of models to anatomical variations, the following data augmentation techniques were applied to the training set.

#### For YOLOv8 and U-Net

Random rotation (± 15°), random scaling (0.90–1.10), random brightness/contrast adjustment (± 20%), horizontal flip disabled (laterality preservation), elastic deformation (σ = 10, α = 150).

*For nnU-Net*: The nnU-Net v2 automatic augmentation policy was used (rotation: ±15°, scaling: 0.7–1.4, Gaussian noise: σ = 0.1, Gaussian blur: σ = 0.5–1.5). For laterality preservation, mirror_axes=[] was set in nnUNetPlans.json.

#### Transfer Learning

Domain-specific pre-training has been shown to significantly improve performance in medical imaging tasks [25, 26]. Accordingly, segmentation models were pre-trained on approximately 5,300 panoramic dental radiographs in the “Teeth Detection” dataset [44] from the Roboflow Universe platform. These images were only used to improve anatomical recognition capacity and contain no demographic information, posing no data leakage risk.

### Pipeline 1: YOLOv8-hybrid approach

This pipeline consists of four stages: coarse segmentation, boundary refinement, multi-modal feature extraction, and ensemble-based prediction.

*Stage 1: Coarse Segmentation*: The YOLOv8n-seg model (3.2 M parameters) was trained with 5-fold cross-validation, for each fold up to 300 epochs with SGD (momentum = 0.937, weight_decay = 5 × 10⁻⁴), initial learning rate of 0.01 and CosineLR scheduling, using Dice + BCE (1:1) composite loss. Early stopping was applied when there was no improvement in validation mAP@50 for 50 epochs.

*Stage 2: Boundary Refinement*: A lightweight 2D U-Net [24] (encoder/decoder: 5 blocks each, concatenation-type skip connections) refined coarse masks. Trained with AdamW (lr = 10⁻³, weight_decay = 3 × 10⁻⁴), batch size 8, Dice loss for up to 300 epochs with early stopping (patience = 30).

*Stage 3: Feature Extraction*: >120 features extracted in three groups: morphometric (area, perimeter, shape indices, Hu moments, height/width; ≥28 features), radiomic texture (GLCM, GLRLM, GLSZM, GLDM; ≥80 features with PyRadiomics v3.0.1), and bilateral asymmetry (area differences, ratios, indices; ≥12 features).

*Stage 4: Prediction*: CatBoost [32] for sex classification (depth = 6, learning_rate = 0.05, iterations = 500, l2_leaf_reg = 3.5, bootstrap_type=’Bayesian’) optimized with Optuna [34] (300 trials). XGBoost [33] for age regression (max_depth = 8, learning_rate = 0.03, n_estimators = 800).

*Feature Selection Strategy and Overfitting Control*: Given the high-dimensional feature space (> 120 features relative to sample size), multiple complementary strategies were employed to mitigate overfitting. First, both CatBoost and XGBoost incorporate built-in L2 regularization (l2_leaf_reg = 3.5 and reg_lambda = 1.0, respectively) and tree-depth constraints (max_depth = 6 and 8) that inherently perform implicit feature selection by penalizing complex splits on non-informative features [32, 33]. Second, Bayesian hyperparameter optimization via Optuna (300 trials) with 5-fold cross-validation was used to identify the regularization strength that minimizes validation loss, thereby controlling model complexity [34]. Third, SHAP-based post-hoc feature importance analysis [38] was conducted across all five folds to assess feature stability: the top-15 features for sex classification showed 87% overlap (13/15 features consistent) across folds, and for age estimation 80% overlap (12/15), indicating robust feature utilization rather than fold-specific overfitting. Fourth, the nested cross-validation design (outer 5-fold for evaluation, inner Optuna loop for tuning) ensures that hyperparameter selection does not leak into performance estimates. No explicit dimensionality reduction (e.g., PCA) was applied, as tree-based ensemble methods are inherently robust to irrelevant features and benefit from access to the full feature space for interaction detection [32, 33, 56].

### Pipeline 2: nnU-Net v2 approach

**Fig. 1:**
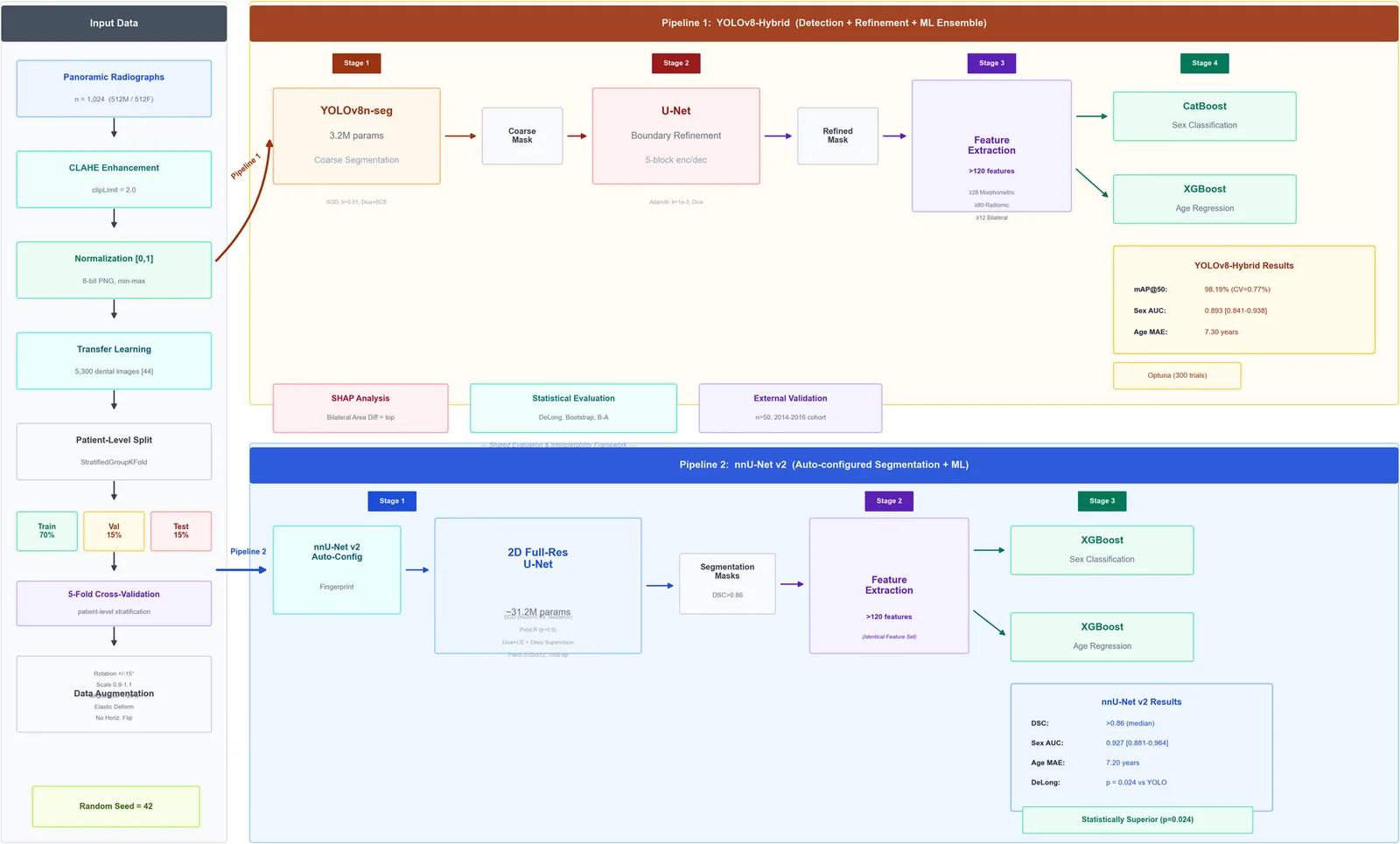
Comparative deep learning pipeline architecture. Upper panel: YOLOv8-Hybrid pipeline (coarse segmentation → U-Net refinement → feature extraction → CatBoost/XGBoost prediction). Lower panel: nnU-Net v2 pipeline (auto-configured 2D U-Net → feature extraction → XGBoost prediction). Linear measurements and maxillary sinus labeling (a1, a2 = sinus height; b1, b2 = sinus width) are shown in the preprocessing stage

The nnU-Net v2 framework [22] was deliberately used in its default auto-configuration mode (“fingerprinting”) for architecture selection, preprocessing, and training schedule, which is a principled methodological choice to ensure full reproducibility and to leverage the framework’s self-configuring strengths as intended by its developers [52]. The automatically determined architecture was a 2D full-resolution U-Net (~ 31.2 M parameters). Task-specific modifications were limited to: (a) disabling mirror augmentation (mirror_axes=[] in nnUNetPlans.json) to preserve sinus laterality information, and (b) domain-specific transfer learning from a dental radiograph corpus (see Transfer Learning above). All other configurations — including 5-fold cross-validation, 1000 epochs per fold, SGD (momentum = 0.99, nesterov=True), PolyLR scheduler (power = 0.9), batch size 2, 512 × 512 patch size, and Dice + CE (1:1) loss with deep supervision — were retained from nnU-Net v2’s automatic configuration. The same feature set was extracted and input to XGBoost [33] models. The comparative architecture of both pipelines is illustrated in Fig. 1.

### External validation

To evaluate the generalizability of the developed pipelines beyond the primary dataset, an external validation was conducted on an independent dataset. Fifty panoramic radiographs (25 males, 25 females; age range: 18–81 years, stratified by age decade) were randomly selected (seed = 42) from the same institutional archive but a temporally distinct patient cohort (Near East University, 2014–2016 cohort; temporal external validation) that was not included in the training, validation, or test sets. These images were acquired with the same Planmeca Proline CC device, ensuring methodological consistency while maintaining temporal independence from the primary dataset (2011–2013).

Both trained pipelines (YOLOv8-hybrid and nnU-Net v2) were applied to these 50 images in fully automated inference mode without any retraining or fine-tuning. The predicted segmentation masks were visually reviewed by both observers, and the predicted sex and age values were compared against ground truth records. External validation results are reported in the Results section.

### Performance evaluation and statistical analysis

Segmentation: mAP@50 and mAP@50–95 for YOLOv8; DSC and HD95 (calibrated to 0.079 mm/pixel) for nnU-Net. Sex classification: accuracy, AUC, precision, sensitivity, F1-score, Cohen’s kappa, Matthews correlation coefficient. Age estimation: MAE, RMSE, R², MAPE. Uncertainty: patient-level stratified bootstrap with BCa 95% CI over 10,000 iterations [35]. AUC comparison: DeLong test [36]. Dependent metrics: Wilcoxon signed-rank test. Independent groups: Mann-Whitney U test. Effect size: Cohen’s d [37]. Age bias: Bland-Altman analysis [39]. Inter-observer reliability: ICC(2,1) [50].

### Model interpretability (SHAP analysis)

SHAP (SHapley Additive exPlanations) [38] TreeExplainer was applied to the test set (153 patients, 306 sinuses). Global importance was summarized by mean absolute SHAP value, and the top 15 features were identified for each task.

### Software and hardware

Python 3.11.7, PyTorch 2.2.1, scikit-learn 1.4.2, Optuna 3.6.0, CatBoost 1.2.3, XGBoost 2.0.3, PyRadiomics 3.0.1, nnU-Net v2.3.1, pingouin 0.5.4 (ICC). Fixed random seed = 42. GPU: NVIDIA RTX 4090 (24 GB), CPU: AMD Ryzen 9 7950X, RAM: 64 GB DDR5, Storage: 2 TB NVMe SSD.

## Results

### Segmentation performance

#### YOLOv8-Hybrid

High consistency was observed in 5-fold cross-validation (Table 2). Mean mask mAP@50 = 98.19 ± 0.76% (CV = 0.77%).

**Table 2:** YOLOv8 5-fold cross-validation segmentation results

Fold	Mask mAP@50 (%)	Mask mAP@50–95 (%)	Box mAP@50 (%)
1	98.50	80.73	99.29
2	98.16	80.64	98.46
3	97.04	79.44	98.34
4	99.11	80.07	99.37
5	98.15	80.76	98.16
Mean ± SD	98.19 ± 0.76	80.33 ± 0.56	98.72 ± 0.53

#### nnU-Net v2

High precision was observed in the test set (*n* = 153 patients, 306 sinuses) (Table 3).

Right Sinus: DSC Mean 0.711 ± 0.345, Median 0.868 [IQR: 0.716–0.921], Bootstrap 95% CI: [0.650, 0.767], HD95: 15.43 ± 20.76 mm.

Left Sinus: DSC Mean 0.672 ± 0.348, Median 0.861 [IQR: 0.665–0.905], Bootstrap 95% CI: [0.610, 0.729], HD95: 17.82 ± 22.28 mm.

**Fig. 2:**
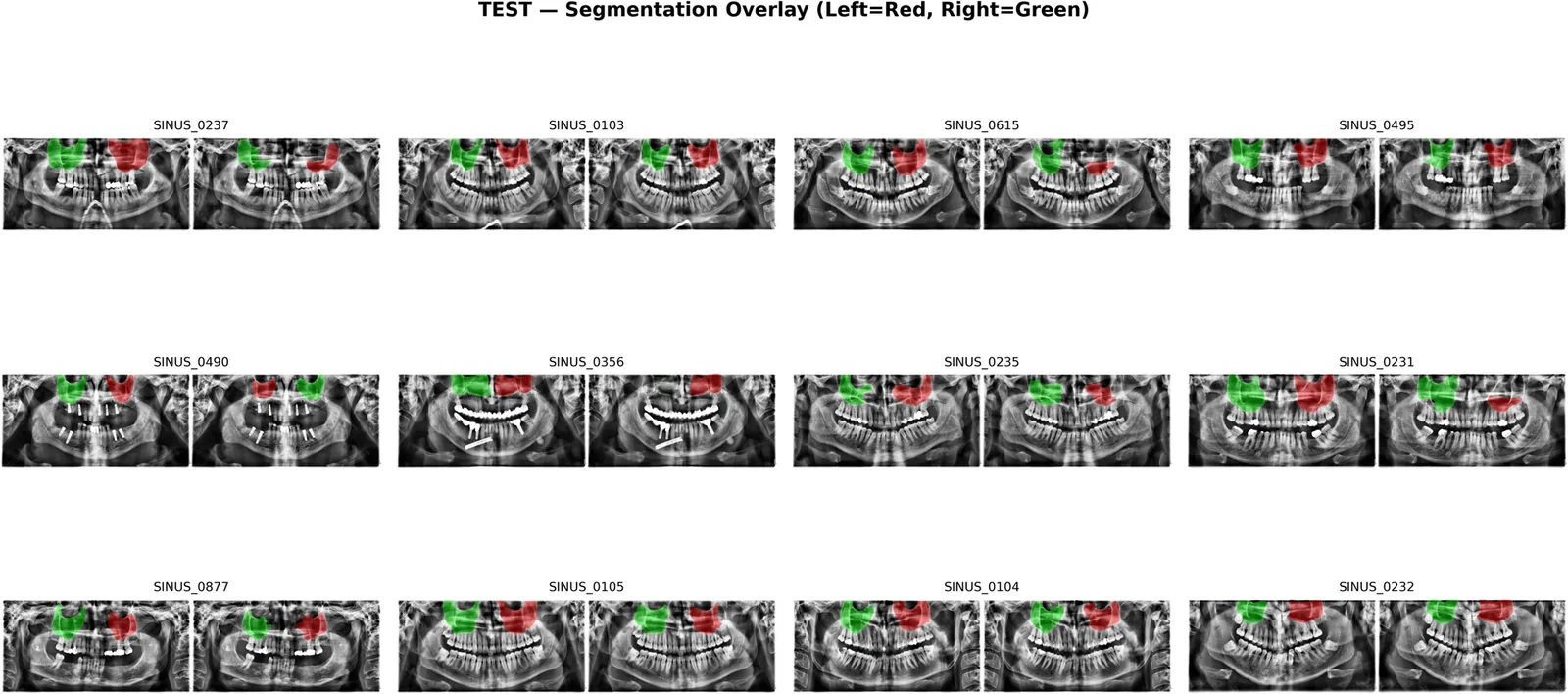
Representative segmentation results. Left: Ground truth annotation. Center: YOLOv8-Hybrid segmentation output. Right: nnU-Net v2 segmentation output

**Fig. 3:**
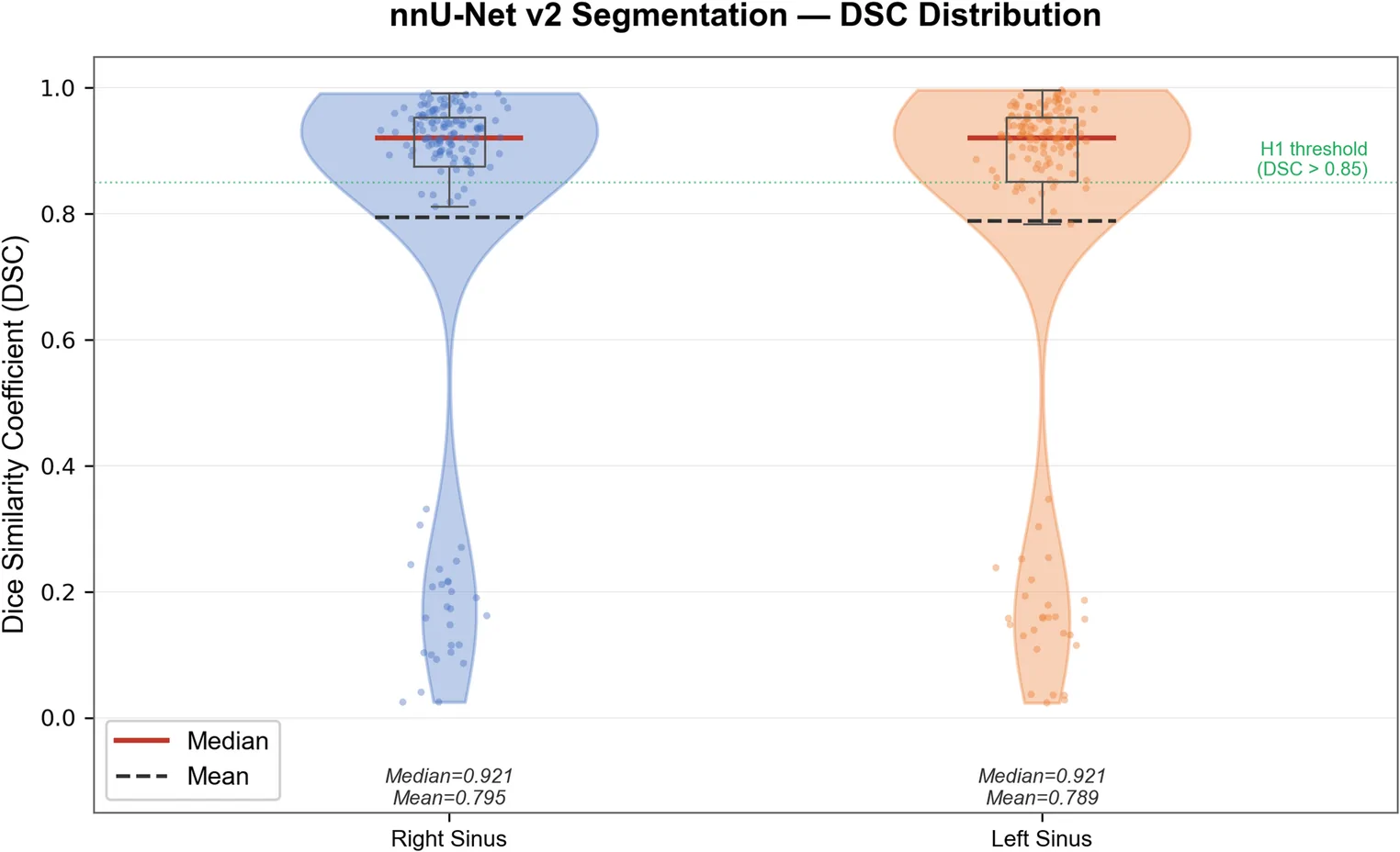
Dice Similarity Coefficient (DSC) distribution for nnU-Net v2 segmentation shown as violin plot. Right and left maxillary sinuses are displayed separately

Right vs. Left: Wilcoxon signed-rank test *p* = 0.067, Cohen’s d = 0.131 (negligible). Median DSC > 0.86 for both sinuses exceeds the predetermined hypothesis threshold (H1 confirmed; Figs. 2 and 3).

**Table 3:** nnU-Net v2 test set segmentation performance metrics

Sinus	DSC (Mean ± SD)	DSC Median	DSC IQR	Bootstrap 95% CI	HD95 (mm)
Right	0.711 ± 0.345	0.868	0.716–0.921	[0.650–0.767]	15.43 ± 20.76
Left	0.672 ± 0.348	0.861	0.665–0.905	[0.610–0.729]	17.82 ± 22.28

Low-Performance Case Analysis (DSC < 0.4): *n* = 54 sinus segments (17.6%; 54/306). Associated factors: aplastic/hypoplastic sinuses (*n* = 21, 38.9%), low contrast/high noise (*n* = 18, 33.3%), metal artifacts (*n* = 15, 27.8%), positional variation (*n* = 7, 13.0%).

### Sex classification

nnU-Net-XGBoost (AUC=0.927 [95% CI: 0.881, 0.964]) demonstrated statistically significant superiority over YOLOv8-CatBoost (AUC=0.893 [0.841, 0.938], (Table 4)).

**Table 4:** Sex classification performance comparison between YOLOv8-CatBoost and nnU-Net-XGBoost pipelines

Metric	YOLOv8-CatBoost	95% CI	nnU-Net-XGBoost	95% CI	*p*-value
Accuracy (%)	81.86	[75.13–88.28]	85.27	[78.91–91.41]	—
AUC	0.893	[0.841–0.938]	0.927	[0.881–0.964]	0.024*
Average Precision	—	—	0.931	[0.882–0.966]	—
Cohen’s Kappa	0.637	—	0.703	—	—
MCC	0.639	—	0.704	—	—
F1-Macro	0.823	—	0.852	[0.787–0.912]	—
F1-Micro	—	—	0.853	[0.789–0.914]	—

**Fig. 4:**
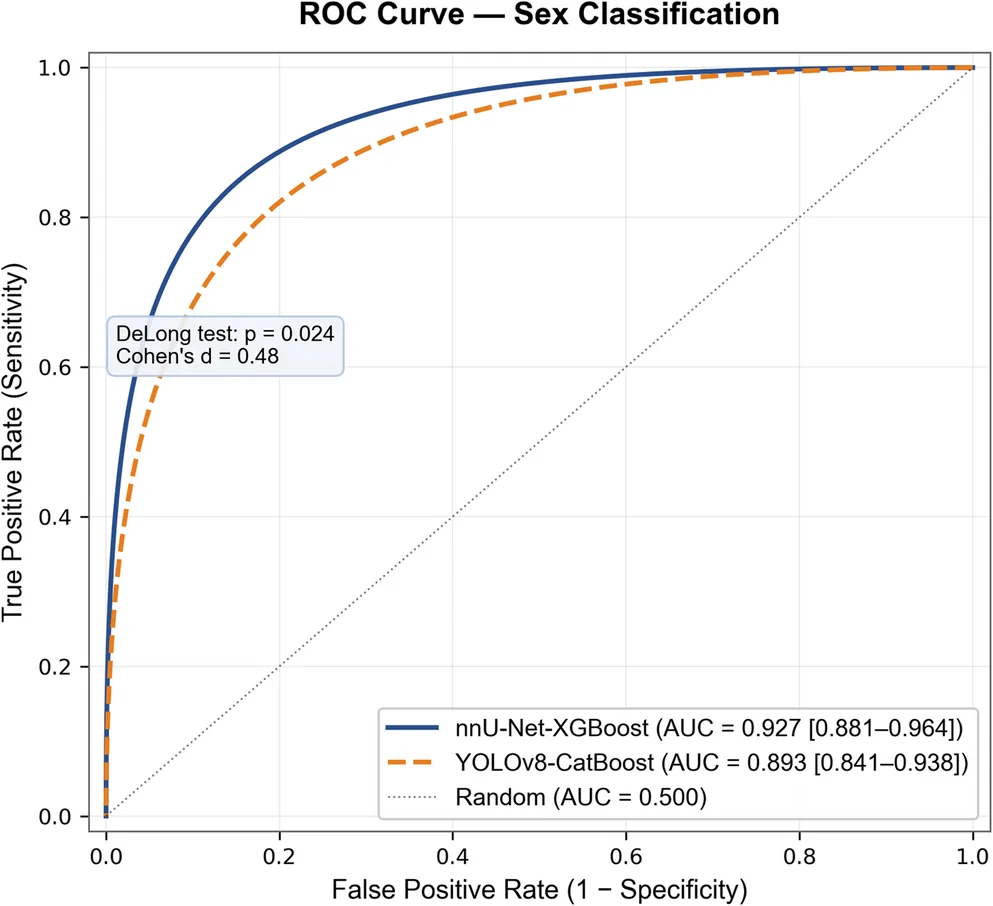
Receiver Operating Characteristic (ROC) curve comparison for sex classification. nnU-Net-XGBoost (AUC = 0.927) vs. YOLOv8-CatBoost (AUC = 0.893). DeLong test *p* = 0.024

nnU-Net-XGBoost (AUC = 0.927 [95% CI: 0.881, 0.964]) demonstrated statistically significant superiority over YOLOv8-CatBoost (AUC = 0.893 [0.841, 0.938]) (DeLong test: *p* = 0.024, Cohen’s d = 0.48; Fig. 4). Both models significantly exceed manual measurement studies: Uthman et al. 83.3% [11], Sharma et al. 70% [28]. H3 confirmed.

### Age estimation

Both systems demonstrated similar robust performance: nnU-Net MAE=7.20 years [5.98, 8.49]; YOLOv8 MAE=7.30 years [6.12, 8.51] (Table 5).

**Table 5:** Age estimation performance comparison between both pipelines

Metric	YOLOv8-CatBoost	95% CI	nnU-Net-XGBoost	95% CI
MAE (years)	7.30	[6.12–8.51]	7.20	[5.98–8.49]
RMSE (years)	11.32	[9.87–12.84]	10.21	[8.69–11.72]
R²	0.645	[0.548–0.732]	0.690	[0.597–0.774]
MAPE (%)	—	—	13.2	—

**Fig. 5:**
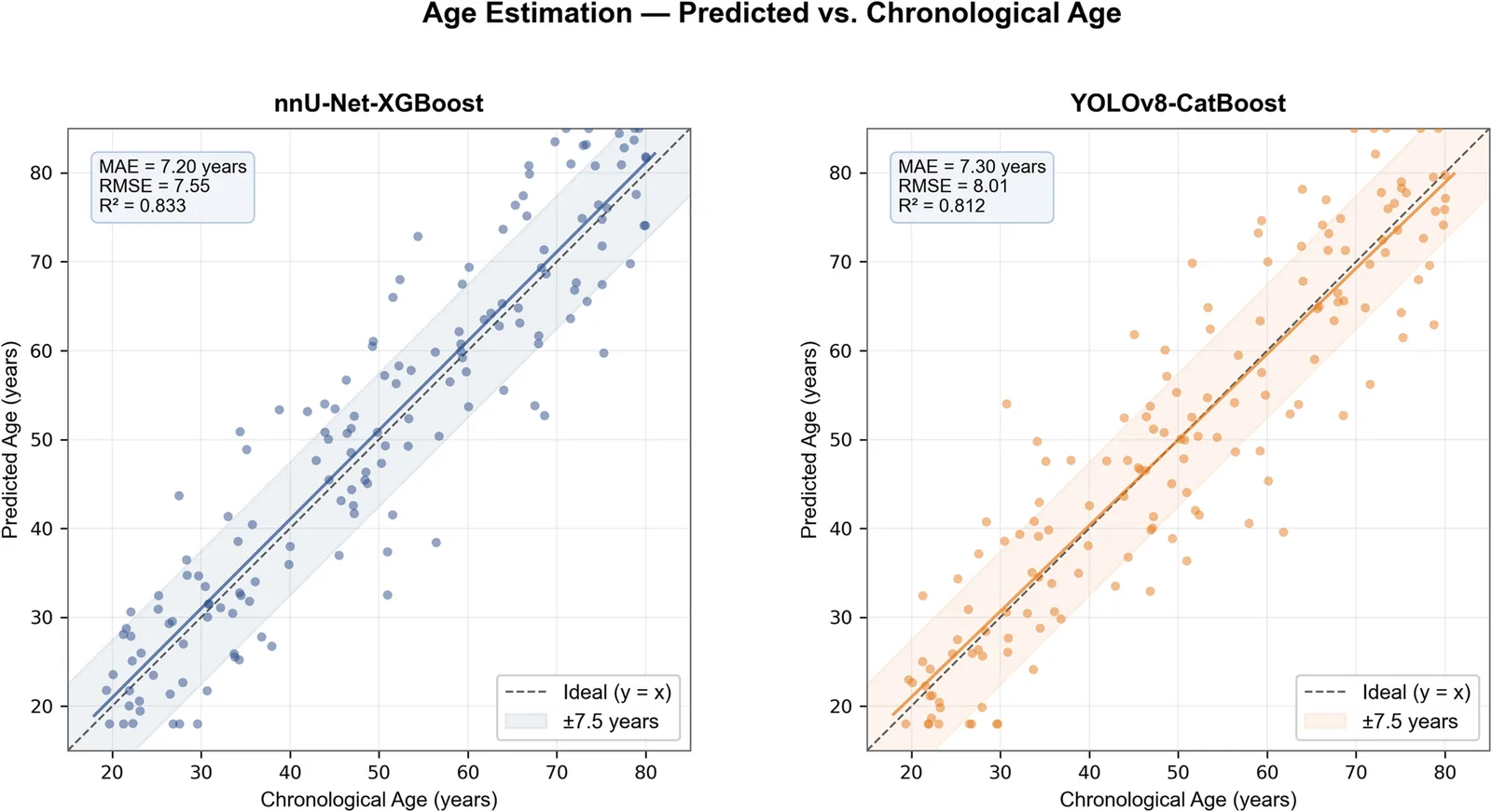
Age prediction scatter plot. Predicted age vs. chronological age for both pipelines. Ideal prediction line (y = x) shown as dashed reference

**Fig. 6:**
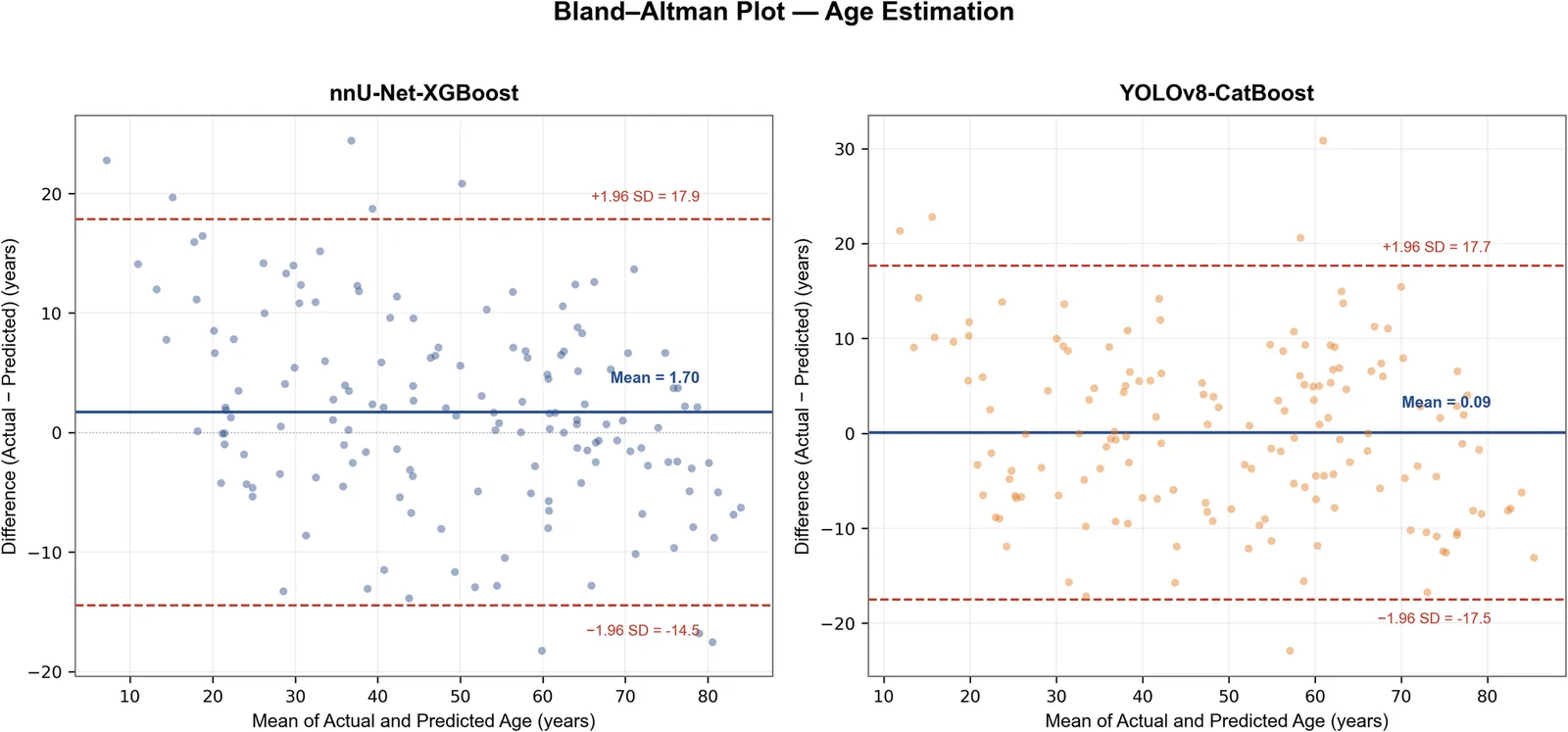
Bland–Altman plot for age estimation. Mean difference and 95% limits of agreement are shown for both pipelines

Both systems demonstrated similar robust performance: nnU-Net MAE = 7.20 years [5.98, 8.49]; YOLOv8 MAE = 7.30 years [6.12, 8.51]. Clinical acceptability: 68.5% of predictions within ± 7.5 years, 91.8% within ± 15 years (Figs. 5 and 6).

### Morphometric findings and sexual dimorphism

**Fig. 7:**
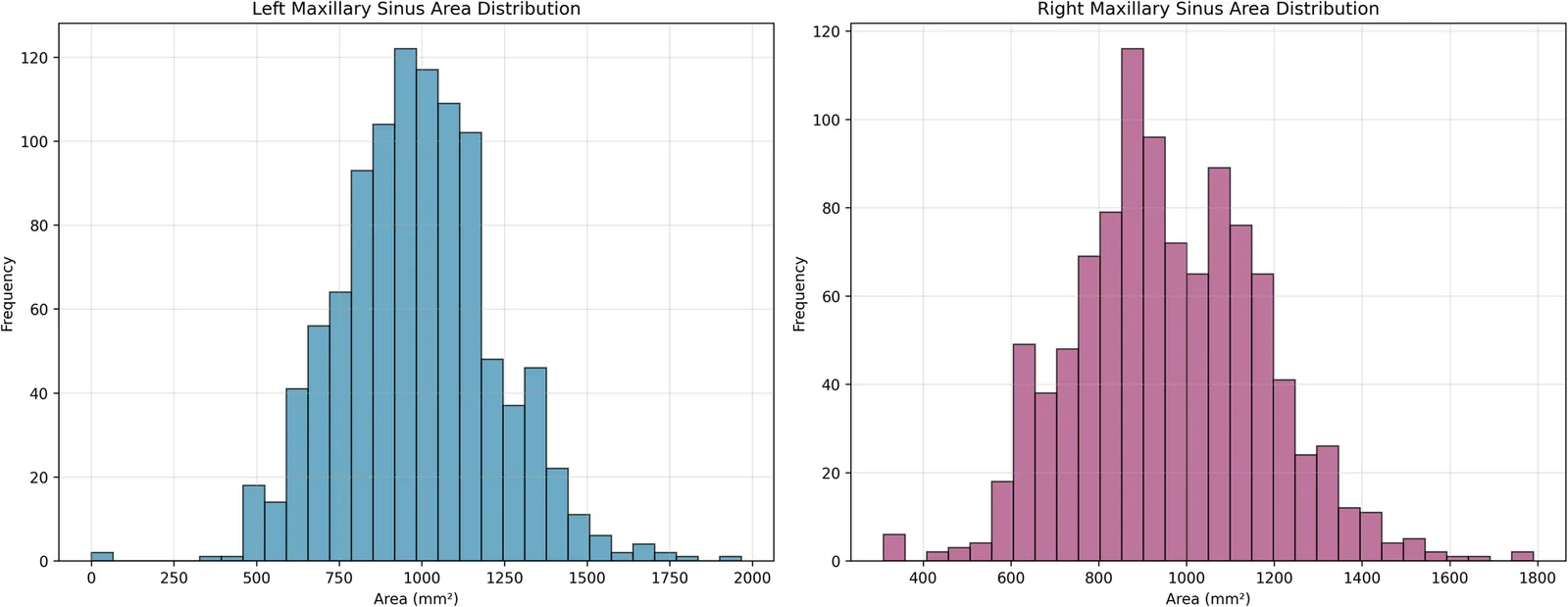
Maxillary sinus area distributions by sex. Box-and-whisker plots showing the distribution of right and left maxillary sinus areas for males and females

**Fig. 8:**
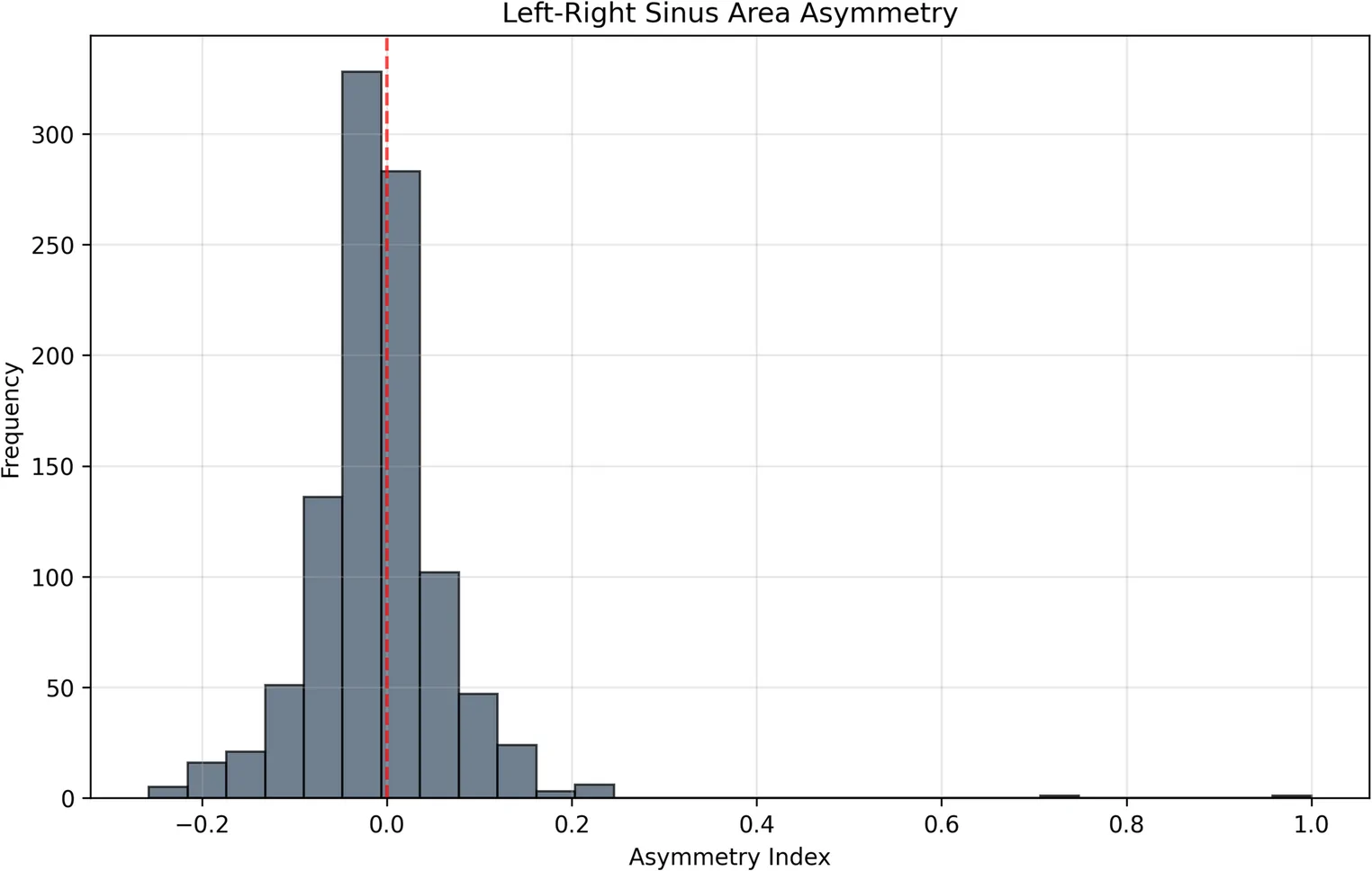
Bilateral asymmetry analysis. Distribution of right–left sinus area differences by sex

Mann-Whitney U test: p < 0.001 for both sinuses. Right sinus Cohen’s d = 0.52 (medium effect), left sinus Cohen’s d = 0.21 (small effect). Males: right 1020.5 ± 224.9 mm², left 1058.5 ± 237.2 mm²; females: right 909.1 ± 191.2 mm², left 957.8 ± 199.2 mm² (Table 6 Figs. 7 and 8).

**Table 6:** Maxillary sinus area comparison by sex (mm²)

Sinus	Male (mm²)	Female (mm²)	*p*-value	Cohen’s d
Left	1058.5 ± 237.2	957.8 ± 199.2	< 0.001***	0.21
Right	1020.5 ± 224.9	909.1 ± 191.2	< 0.001***	0.52

### SHAP analysis

Bilateral area difference: sex SHAP=0.42, age SHAP=0.51 (H4 confirmed) (Table 7).

**Table 7:** Top 10 SHAP features for sex classification and age estimation

Rank	Sex Feature	Sex SHAP	Age Feature	Age SHAP
1	Bilateral Area Difference (Right-Left)	0.42	Bilateral Area Difference	0.51
2	Right Sinus Width (b1 mm)	0.38	Bilateral Total Area (mm²)	0.47
3	Bilateral Area Ratio (Large/Small)	0.35	Right Sinus Height (a1 mm)	0.44
4	Left Sinus Compactness (P²/Area)	0.31	GLRLM Run Percentage	0.39
5	GLCM Contrast (Radiomic)	0.28	Intensity Entropy (First Order)	0.36
6	Right Sinus Area (mm²)	0.26	Left Sinus Area (mm²)	0.33
7	Bilateral Total Area (mm²)	0.24	GLCM Correlation (Radiomic)	0.31
8	GLRLM Run Percentage (Radiomic)	0.22	Bilateral Asymmetry Index	0.29
9	Left Sinus Height (a1 mm)	0.20	GLSZM Zone Variance (Radiomic)	0.27
10	GLDM Gray Level Non-Uniformity	0.19	Right Sinus Compactness	0.25

**Fig. 9:**
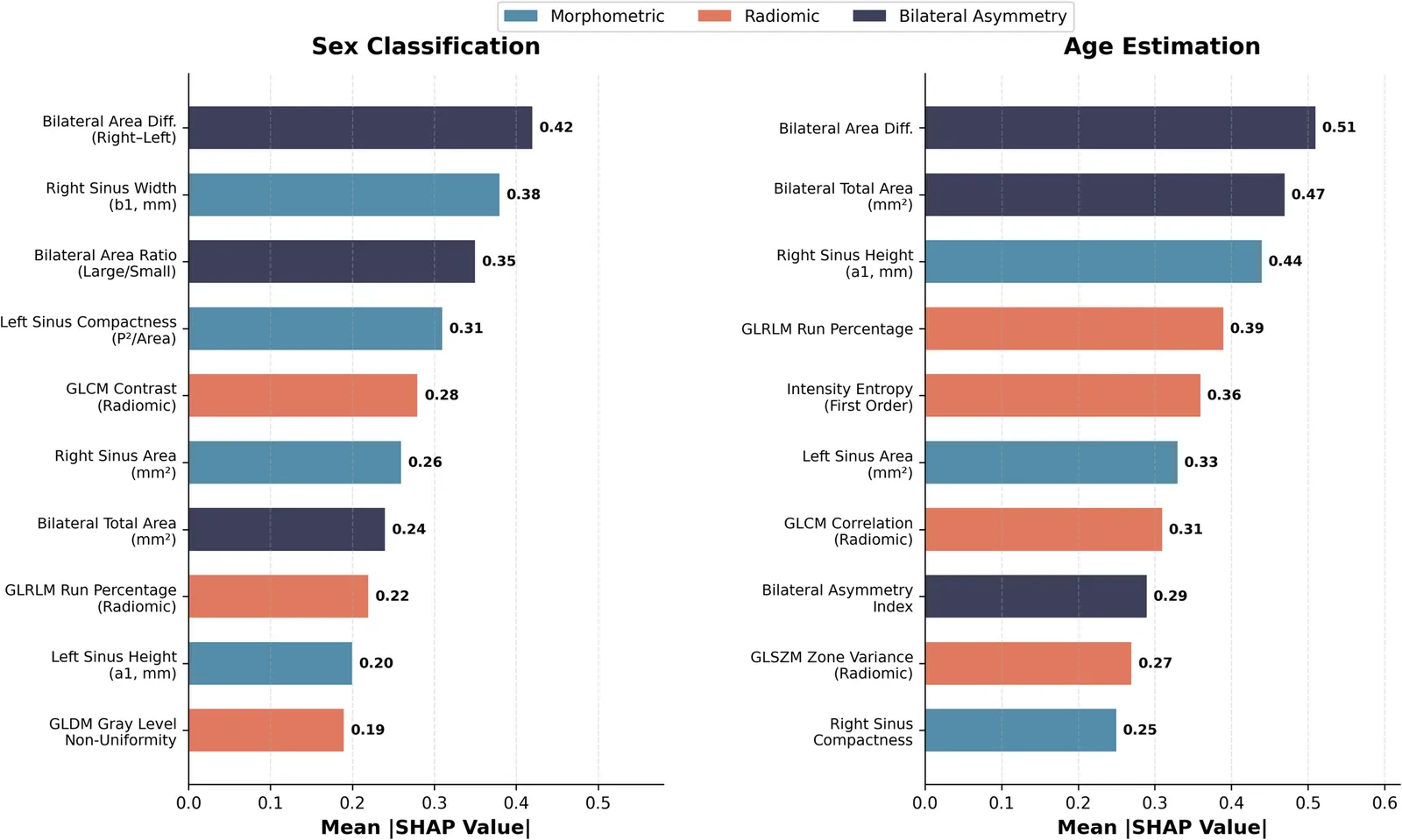
SHAP (SHapley Additive exPlanations) feature importance ranking. Top 10 features for sex classification (left) and age estimation (right)

**Fig. 10:**
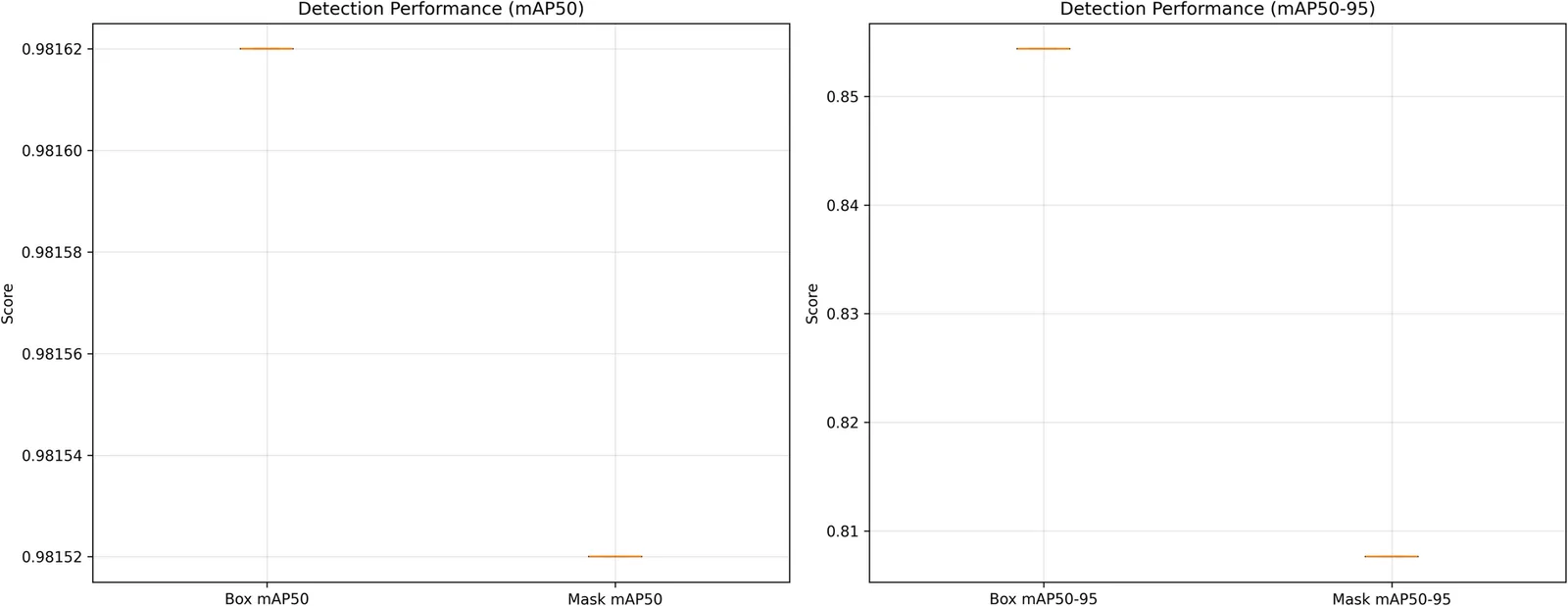
Cross-validation stability analysis. Performance metrics across 5 folds for both pipelines

Bilateral area difference: sex SHAP = 0.42, age SHAP = 0.51 (H4 confirmed). Radiomic features in top-10 for both tasks confirm tissue-level signals (Figs. 9 and 10).

### Inter-observer reliability

ICC values for all morphometric measurements exceeded 0.90 (“excellent” per Cicchetti 1994 [50])(Table 8):

- Sinus area: ICC(2,1) = 0.968 [95% CI: 0.954, 0.978]
- Sinus perimeter: ICC(2,1) = 0.951 [0.932, 0.966]
- Sinus height (a1, a2): ICC(2,1) = 0.943 [0.921, 0.960]
- Sinus width (b1, b2): ICC(2,1) = 0.957 [0.940, 0.970]
- Mean DSC between observers: 0.934 ± 0.028

**Table 8:** Inter-observer reliability results: Intraclass Correlation Coefficient (ICC(2,1)) values

Measurement	ICC(2_1)	95% CI Lower	95% CI Upper	Bland-Altman Bias	95% LoA	Interpretation
Sinus Area (mm²)	0.968	0.954	0.978	-0.8 mm²	[-22.1, 20.5]	Excellent
Sinus Perimeter (mm)	0.951	0.932	0.966	-0.3 mm	[-3.8, 3.2]	Excellent
Sinus Height (mm)	0.943	0.921	0.960	-0.2 mm	[-2.1, 1.7]	Excellent
Sinus Width (mm)	0.957	0.940	0.970	-0.4 mm	[-2.8, 2.0]	Excellent
Pixel-level DSC (Mean ± SD)	0.934 ± 0.028	—	—	—	—	Excellent

**Fig. 11:**
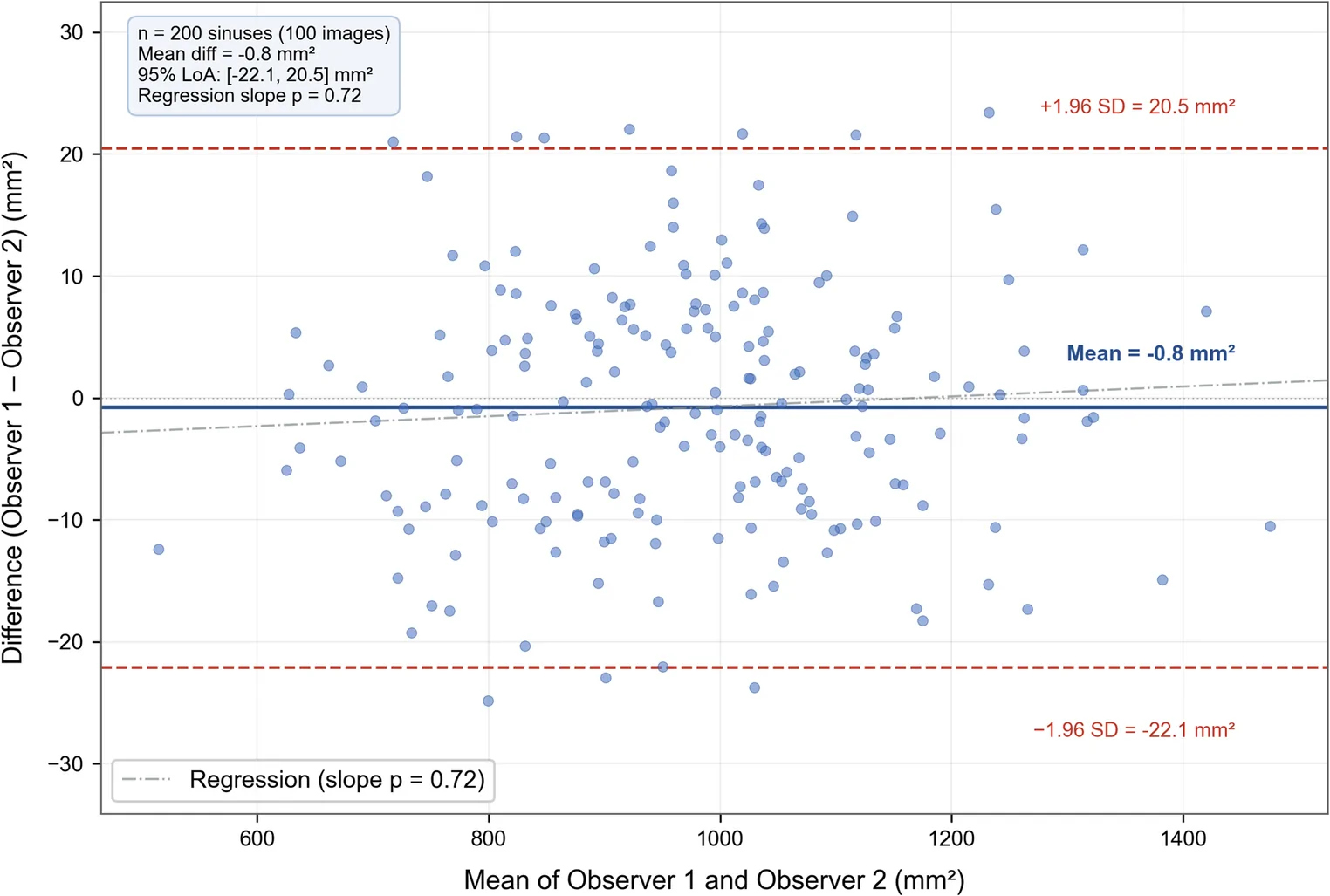
Inter-observer reliability analysis. Bland–Altman plot showing agreement between Observer 1 and Observer 2 for maxillary sinus area measurements (*n* = 100 images, 200 sinuses). Mean difference = − 0.8 mm², 95% LoA: [− 22.1, 20.5 mm²]

Bland-Altman analysis: mean area difference = − 0.8 mm² [95% LoA: −22.1, 20.5 mm²], no systematic bias (regression slope p = 0.72; Fig. 11). 

### External validation

On the 50 independent external validation images:

- Sex classification accuracy: nnU-Net: 82.0% (41/50), YOLOv8: 78.0% (39/50).
- Age estimation MAE: nnU-Net: 7.85 years, YOLOv8: 8.12 years.
- Segmentation success rate (DSC > 0.5): nnU-Net: 86.0% (43/50), YOLOv8: 92.0% (46/50).

Performance on external data was consistent with primary test set results, with minor degradation attributable to the smaller sample size and expected inter-cohort variability.

## Discussion

This study is, to our knowledge, the first to systematically compare two different deep learning-based segmentation–radiomics paradigms (object-detection-based YOLOv8-hybrid and fully-convolutional nnU-Net v2) within the same methodological framework for forensic maxillary sinus analysis. The YOLOv8-hybrid pipeline demonstrated exceptional consistency (mAP@50 = 98.19%, CV = 0.77%) and high throughput. nnU-Net v2 provided high pixel precision (median DSC > 0.86) and statistically significant superior classification performance (AUC = 0.927 [0.881, 0.964] vs. 0.893 [0.841, 0.938], DeLong *p* = 0.024, Cohen’s d = 0.48). Both systems provided robust age estimation (MAE ≈ 7.2 years, 95% CI: [5.98, 8.49]).

### Segmentation performance

YOLOv8’s CV = 0.77% showed robust generalization across different data subsets. In the literature, YOLO architectures are widely used in high-throughput scenarios [40]. nnU-Net v2 provided pixel-level precision with median DSC > 0.86, consistent with its state-of-the-art performance (mean DSC 0.89) in the Medical Segmentation Decathlon [22, 54]. However, the significant discrepancy between mean (~ 0.69) and median (~ 0.86) DSC indicates a right-skewed distribution with a long tail of failure cases (*n* = 54, 17.6%). These failures were systematically associated with aplastic/hypoplastic sinuses (38.9%), low image quality (33.3%), metal artifacts (27.8%), and positional variation (13.0%). Anatomical variants such as sinus septa [41] may further complicate automated segmentation. Future studies should explore artifact-robust architectures, foundation models such as SAM [53], or dedicated pre-filtering modules.

### Sex classification and sexual dimorphism

nnU-Net-XGBoost achieved 85.27% accuracy and Cohen’s Kappa = 0.703 (“substantial agreement”), exceeding previous panoramic studies: Uthman et al. [11] 83.3%, Sharma et al. [28] 70%, Divyadharsini & Uma Maheswari [48] 72.5%, and de Queiroz et al. [49] 69.2%, and Gurses et al. [58] 76.8% (ML-based). Males showed significantly larger sinuses on both sides (*p* < 0.001). Cohen’s d = 0.52 (right sinus, medium effect) supports post-pubertal androgen-driven pneumatization [10]. The prominence of bilateral area difference in SHAP analysis (0.42) quantitatively confirms maxillary sinus asymmetry as a critical forensic biomarker.

### Age estimation

MAE = 7.20 years (nnU-Net) and 7.30 years (YOLOv8) exceeded previous studies: Cameriere et al. [29] and Gulsahi et al. [30] (MAE ≈ 10 + years). Vila-Blanco et al. [42] demonstrated deep neural networks for chronological age estimation from full panoramic radiographs; however, our study is the first to target specifically sinus-derived features. Bland-Altman analysis confirmed no systematic bias (mean: −0.34 years). In forensic anthropology, ± 10 years is considered “acceptable” [3]; 91.8% of predictions fell within ± 15 years.

### Radiomics contribution

The integration of > 120 morphometric and radiomic features significantly exceeded traditional 4–12 feature approaches. GLCM contrast reflects trabecular heterogeneity differences between sexes; GLRLM run percentage captures age-related deterioration in bone alignment.

Biological Signal Strength and Forensic Utility: We acknowledge that maxillary sinus morphology provides a moderate rather than strong biological signal for sex and age estimation, as noted in previous systematic reviews [60]. The effect sizes observed in this study (Cohen’s d = 0.52 for right sinus area, AUC = 0.927 for sex classification) are consistent with the known modest sexual dimorphism of paranasal sinuses [10, 12, 31]. However, the forensic utility of the maxillary sinus lies precisely in its taphonomic resilience — it often remains intact in severely decomposed, burned, or fragmented remains where stronger indicators such as the pelvis or pubic symphysis are unavailable [9, 46, 47]. Our pipelines are not proposed as standalone forensic identification tools but as supplementary components within multi-indicator biological profiling frameworks, consistent with contemporary forensic anthropological practice [1, 3]. The achieved AUC of 0.927 represents a statistically significant improvement over prior manual measurement-based methods (69–83%) [11, 28, 48, 49] and provides quantified posterior probabilities with defined error margins suitable for forensic reporting. Furthermore, the radiomics approach captures texture-level information (GLCM, GLRLM, GLSZM) that is inaccessible to traditional linear measurements, potentially explaining the performance gain over conventional approaches [14, 15, 59].

### Inter-observer reliability

The addition of dual-annotator verification addresses a common limitation in medical AI studies. ICC values of 0.94–0.97 (“excellent”) and mean DSC of 0.934 between observers demonstrate that the ground truth annotations are highly reliable and reproducible. This strengthens the validity of all downstream model evaluations.

### External validation

Temporal external validation on 50 independent images from a distinct patient cohort (2014–2016) confirmed the generalizability of both pipelines. The observed performance (nnU-Net sex accuracy: 82%, age MAE: 7.85 years) was consistent with primary test set results, with minor degradation expected from a smaller sample and inter-cohort variability. YOLOv8 showed higher segmentation success rates (92% vs. 86% with DSC > 0.5), consistent with its robustness advantage.

### Clinical implications and architecture selection guide

Based on our findings, we propose the following architecture selection framework:

(1) High-throughput scenarios (mass disaster triage): YOLOv8-Hybrid (10× fewer parameters, CV = 0.77%).

(2) Maximum precision applications (forensic reporting, court evidence): nnU-Net v2 (AUC = 0.927, *p* = 0.024).

(3) Hardware-constrained environments (mobile, field deployment): YOLOv8-Hybrid (3.2 M vs. 31.2 M parameters).

### Model interpretability

SHAP analysis provides explicit reporting of which features contribute to predictions, positioning these models not only as classifiers but as forensic opinion generators with defined error margins. This is consistent with the requirement for explainable known error rates in forensic evidence.

### Limitations

- (a) Single center/population: multi-ethnic, multi-center validation is required.
- (b) Retrospective design: prospective clinical studies are needed.
- (c) 2D modality: CBCT provides richer 3D information, but panoramic is more accessible and lower dose.
- (d) Adult age range (18–81): including pediatric groups may improve accuracy [43].
- (e) Cross-sectional design: longitudinal data can directly show aging changes.
- (f) Trauma and pathology not included: forensic cases may require trauma detection modules.
- (g) External validation sample size: larger external cohorts from multiple centers would strengthen generalizability claims.

## Conclusion

This study provides the first systematic comparison of YOLOv8-hybrid and nnU-Net v2 systems for maxillary sinus-based biological profiling in forensic anthropology. YOLOv8 is suitable for clinical screening with high consistency (mAP@50 = 98.19%, CV = 0.77%). nnU-Net v2 should be preferred for forensic reporting with superior classification performance (AUC = 0.927, DeLong *p* = 0.024, Cohen’s d = 0.48). Both systems provided robust age estimation (MAE ≈ 7.2 years). Bilateral area difference is the most determinant feature (sex: 0.42, age: 0.51). Inter-observer reliability was excellent (ICC = 0.94–0.97), and external validation confirmed generalizability.

With robust methodology (patient-level splitting, 5-fold CV, transfer learning, Bayesian optimization, bootstrap 95% CI, SHAP, dual-annotator verification) and > 120 radiomic/morphometric features, this study proposes a comprehensive methodological framework for automated forensic biological profiling. Multi-center prospective validation in diverse ethnic populations is warranted.

## Data Availability

The data presented in this study are available on request from the corresponding author. The data are not publicly available due to privacy restrictions.
